# Impact of prostate weight on perioperative outcomes of robot-assisted laparoscopic prostatectomy with a posterior approach to the seminal vesicle

**DOI:** 10.1186/1471-2490-14-6

**Published:** 2014-01-09

**Authors:** Takahiro Yasui, Keiichi Tozawa, Satoshi Kurokawa, Atsushi Okada, Kentaro Mizuno, Yukihiro Umemoto, Noriyasu Kawai, Shoichi Sasaki, Yutaro Hayashi, Yoshiyuki Kojima, Kenjiro Kohri

**Affiliations:** 1 Department of Nephro-urology, Nagoya City University Graduate School of Medical Sciences, 1 Kawasumi, Mizuho-cho, Mizuho-ku, 467-8601, Nagoya, Japan; 2 Department of Urology, Fukushima Medical University School of Medicine, Fukushima, Japan

**Keywords:** Median lobe, Prostate cancer, Radical prostatectomy, Robot-assisted surgery

## Abstract

**Background:**

To determine the effect of prostate weight on the preoperative and postoperative outcomes of robotic-assisted laparoscopic radical prostatectomy with a posterior approach to the seminal vesicle.

**Methods:**

This retrospective study examined prospectively collected data on 219 robotic-assisted laparoscopic radical prostatectomies performed from May 2011 to February 2013. Patients were divided into four groups based on pathologic prostate weight: <30 g, 30–49 g, 50–79 g, and ≥80 g. Continence and sexual function were assessed using validated questionnaires.

**Results:**

Of the 219 patients, 19, 143, 51, and 6 had prostates weighing <30 g, 30–49 g, 50–79 g, and ≥80 g, respectively. Significant differences were found between the preoperative Gleason scores, total operative times, and robotic times of the groups. Both estimated blood loss and anastomosis time tended to be greater in the higher prostate weight groups, but the differences were not significant. No significant differences were observed in transfusion rate, length of catheterization, complication incidence, or positive surgical margins. The return of urinary function, as determined by questionnaire scores, was not affected by prostate weight.

**Conclusions:**

Robotic-assisted laparoscopic radical prostatectomy can be performed safely and with similar perioperative outcomes, regardless of prostate weight. Indeed, oncological outcome, urinary continence, and complications were similar across the prostate weight groups, suggesting that robotic-assisted laparoscopic radical prostatectomy with a posterior approach to the seminal vesicle may be performed effectively on men with large prostates, despite greater surgical times.

## Background

Robot-assisted laparoscopic prostatectomy (RALP) has become a common treatment option for patients with localized prostate cancer [1]. Comparisons of open radical retropubic prostatectomy with RALP have demonstrated that these procedures have nearly equivalent oncological outcomes and similar rates of complications relating to continence and erectile function [2].

The difficulty of dissecting a large median lobe has been recognized since the early history of prostatectomy. Accordingly, the effects of prostate weight on radical prostatectomy have been studied for open radical prostatectomy and laparoscopic radical prostatectomy [3-5]. Zorn et al. [6] reported that prostate size affects oncological outcomes in RALP, but does not affect operative times. In addition, it has been reported that the presence of a large median prostate lobe component increases the difficulty of performing RALP [7], presumably because larger prostates decrease visualization of the surgical field when performing RALP.

In the present study, we assessed RALP with a posterior approach to the seminal vesicle. The posterior approach has the advantage of retaining a good visualization of the prostate and posterior bladder, irrespective of prostate size. To analyze the influence of large prostates on RALP with a posterior approach, we examined the relationship between prostate weight and outcomes for our first 219 patients who underwent this procedure.

## Methods

### Subjects

Between May 2011 and February 2013, 219 consecutive patients were recruited for this study, each of whom underwent RALP at Nagoya City University Hospital. The study received approval from our institutional review board (“Robot-assisted laparoscopic radical prostatectomy for prostate cancer”, No. 46-10-0009) and conforms to the provisions of the Declaration of Helsinki. Each patient provided informed consent. The preoperative assessments of all patients included detailed patient histories, clinical examinations, serum prostate-specific antigen (PSA) measurements, biopsy findings, Gleason score measurements, bone scan results, and contrast-enhanced computed tomography (CT) or magnetic resonance imaging (MRI) findings. Baseline demographic clinical staging was conducted according to the TNM staging system (Union Internationale Contre le Cancer, 2002 classification), and only patients with T_1–3_N_0_M_0_ stage cancers were considered for RALP. Patients were divided according to their pathologic prostate weight as follows: group 1, <30 g; group 2, ≥30 g to <50 g; group 3, ≥50 g to <80 g; and group 4, ≥80 g. Preoperative, intraoperative, and postoperative parameters were analyzed. One patient, who required open conversion, was excluded from all statistical analyses.

The procedures were performed by five surgeons with experience in open radical prostatectomy and laparoscopic radical prostatectomy. The entire surgical team underwent 1 week of intensive training at Sukagawa Training Center (Fukushima, Japan), and received surgical practice at the St. Augustin Hospital (Bordeaux, France) and the Yonsei University Severance Hospital (Seoul, Korea).

The following data were collected and reviewed for the present study: patient age, body mass index (BMI), total operating time (including port placement, docking of the robot, dissection, anastomosis, and closure), duration of robotic surgery, duration of anastomosis, estimated blood loss, presence or absence of urinary incontinence (pad usage), duration of postoperative bladder catheterization, intraoperative complications, immediate postoperative complications (appearing within the first month after surgery), long-term complications (appearing after the first postoperative month), TNM staging, and surgical margins status.

### Surgical technique

All patients eligible for radical prostatectomy were offered RALP, which was performed using a 4-arm da Vinci-S robotic system (Intuitive Surgical, Sunnyvale, USA). Our technique was based on that used at the Vattikuti Institute (Detroit, USA), combined with the use of diathermy scissors [8]. Previously, we had performed laparoscopic radical prostatectomy using a posterior approach to the seminal vesicle and following the Montsouris method [9]. Therefore, we adopted the same approach for the RALP procedure [10]. Briefly, we used an intraperitoneal approach. The seminal vesicles were dissected first, using a posterior approach. The rectum was retracted in a cephalad dissection by the assistant. The superior peritoneal arch (created by the impression of the Foley balloon) was grasped by the assistant or the third arm of the da Vinci-S and lifted upwards. The monopolar scissors were then used to create a curvilinear incision midway between the anterior rectal wall and the grasped arch. The incision was deepened by blunt dissection through the fibro-alveolar tissue, revealing both vas deferens (VDs). The VDs were dissected free, approximately 3 cm from the prostate, and transected. Blunt dissection of the anterior fibrovascular tissue overlying the seminal vesicles (SVs) was continued laterally. Once the dissection had been completed to the level of the base, blunt medial dissection freed the posterior surface of the SVs. After both SVs were completely dissected, upward traction on both SVs and VDs facilitated an incision into Denonvillier’s fascia, which allowed the posterior dissection to continue to the level of the rectourethralis fibers. Subsequently, dorsal vein control was achieved using 2-0 V-Loc™ on a 37-mm needle (Covidien, Mansfield, USA). The vascular pedicles were clipped with Hem-o-lock (Teleflex, Limerick, USA) to control the posterolateral small vessels when nerve sparing was performed. Thereafter, the prostatectomy was conducted in an almost antegrade fashion, preserving the neurovascular bundles when indicated using a clipless technique [11]. The Rocco suture [12] was also adopted, using 3-0 V-Loc™ on a 26-mm needle (Covidien). Large bladder neck defects were reconstructed using a “fish mouth” or “parachute” technique with anastomosis to the urethral stump, as chosen according to the surgeon’s preference [13]. In the “fish mouth” technique, two interrupted Vicryl sutures (from the 2-o’clock to the 4-o’clock position and from the 8-o’clock to the 10-o’clock position) were placed on the bladder neck, to narrow the diameter of the bladder neck opening until it matched the urethral diameter. Finally, we performed running vesicourethral anastomosis using two 3-0 PDSII (Ethicon Endo-Surgery, Cincinnati, USA) sutures, retightened with Lapra-Ty (Ethicon Endo-Surgery) at the 3- and 9-o’clock positions. Each patient who had an intermediate or high D’Amico risk [14] underwent lymph node dissection. Nerve-sparing surgery was performed for the 43 cases in which unilateral cancer was detected by needle biopsy or the patient expressed a desire for nerve-sparing surgery.

### Complications

Intraoperative and postoperative complications were critically assessed and compared across the four prostate weight groups. Postoperative complications were graded according to a system described by Clavien [15]. Following this system, grade I complications include alterations from the ideal postoperative course that are not life threatening and cause no lasting disability. Grade II complications require pharmacological treatment with drugs, blood transfusions, or total parenteral nutrition. Grade III complications require surgical, endoscopic, or radiological intervention. Grade IV complications are life threatening. Grade V is assigned when death results from a complication.

### Pathological analysis of specimens

The final Gleason score, degree of positive margins, and seminal vesicle or lymph node involvement were included in histopathological assessments. The specimen’s pathological processing included 4-mm sectioning of the whole gland. The presence of malignant glands in direct contact with the inked surface was defined as constituting positive margins.

### Patient follow-up

Follow-up visits were conducted at regular intervals. Follow-up included serial PSA monitoring in addition to assessments of functional outcomes, such as continence and erectile function. The rate of continence was investigated for the 199 patients (91%) who had over 6 months of follow-up data. Patients with urge incontinence were excluded from the follow-up study. Continence was assessed using a questionnaire that was provided 1, 3, and 6 months after RALP. Functional data were prospectively assessed using the Expanded Prostate Cancer Index Composite. Men requiring greater than 0 precautionary pads per day were considered incontinent. Twelve months of follow-up data on incontinence were available for 101 patients (46%).

### Statistical analysis

An analysis of variance model was used to compare continuous outcome variables among the groups. Chi-squared and Fisher’s exact tests were used to compare categorical outcome variables. Each significant overall effect was additionally investigated in a post-hoc pairwise comparative analysis that used the Tukey test and Bonferroni-adjusted P values. Two-tailed P values <0.05 were considered statistically significant.

## Results

### Baseline demographics

Patient demographics are presented in Table 1. The overall mean prostate weight was 43.6 g (range, 18–110). Most patients had non-palpable disease that was detectable with MRI (clinical Stage T2). We observed no significant associations between prostate weight and preoperative demographics, which include age, PSA and BMI. In the largest prostate group (>80 g), no patients received nerve-sparing surgeries.

**Table 1:** Perioperative features of RALP patients based on pathologic prostate weight

**Variable**	**<30**	**≥30, <50**	**≥50, <80**	**≥80**	**P Value**
**Patients (n)**	**19**	**143**	**51**	**6**	
Prostate weight (g)	24.5 (18–29)	38.5 (30–48)	58.6 (50–78)	97.7 (86–110)	<0.01
Mean age (y)	65.9 (50–76)	66.5 (49–75)	67.6 (41–75)	67.7 (60–72)	0.63
Mean PSA (ng/mL)	9.7	7.5	9.0	9.9	0.30
BMI	23.7	23.3	23.4	25.3	0.08
Preoperative Gleason score					
5–6	3 (15.8%)	39 (27.3%)	24 (47.1%)	4 (66.7%)	<0.01
7	14 (73.7%)	59 (41.3%)	11 (21.6%)	2 (33.3%)	
8–9	2 (10.5%)	45 (31.5%)	16 (31.4%)	0	
Clinical stage					
T1c	4 (21.1%)	40 (28.0%)	15 (29.4%)	5 (83.3%)	0.34
T2a	4 (21.1%)	38 (26.6%)	16 (31.4%)	1 (16.7%)	
T2b	4 (21.1%)	12 (8.4%)	4 (7.8%)	0	
T2c	7 (36.8%)	44 (30.8%)	14 (27.5%)	0	
T3a	0	8 (5.6%)	1 (2.0%)	0	
T3b	0	1 (0.7%)	1 (2.0%)	0	
Nerve sparing					
None	14 (73.7%)	113 (79.0%)	43 (84.3%)	6 (100.0%)	0.62
Unilateral	3 (15.8%)	24 (16.8%)	5 (9.8%)	0	
Bilateral	2 (10.5%)	6 (4.2%)	3 (5.9%)	0	
Estimated blood loss (mL)	241.8	304.2	259.8	586.0	0.05
Transfusion	1 (5.3%)	6 (4.2%)	2 (3.9%)	0	1.21
Mean total operative time (min)	223.7	219.6	219.3	270.7	<0.01
Mean robotic time (min)	165.4	163.8	162.4	216.5	<0.01
Mean anastomosis time (min)	26.5	25.7	23.0	32.5	0.13
Mean Foley catheterization (days)	7.58	7.76	8.36	8.83	0.54
Pathological stage					
pT2a	1 (5.3%)	18 (12.6%)	14 (27.5%)	4 (66.7%)	<0.01
pT2b	3 (15.8%)	2 (1.4%)	1 (2.0%)	0	
pT2c	13 (68.4%)	96 (67.1%)	29 (56.9%)	2 (33.3%)	
pT3a	1 (5.3%)	11 (7.7%)	5 (9.8%)	0	
pT3b	1 (5.3%)	16 (11.2%)	2 (3.9%)	0	
Positive margins	7/19	54/143	13/51	1/6	0.33
	(36.8%)	(37.8%)	(25.5%)	(16.7%)	
pT2	5/17	39/116	9/44	1/6	0.37
	(29.4%)	(33.6%)	(20.5%)	(16.7%)	
pT3	2/2	15/27	4/7	0/0	0.47
	(100.0%)	(55.6%)	(57.1%)	(0.0%)	
Mean follow-up (mo)	12.0	11.8	10.6	9.7	0.43

### Perioperative outcomes

Transfusion rates and mean Foley catheterizations were similar across the four groups (Table 1). The series excluded one open conversion, which resulted from abdominal adhesion and was one of the first 20 cases in the series. Total operation times and robotic times were significantly greater among patients with prostate weights ≥50 g (P < 0.01). Estimated blood loss and anastomosis time tended to be greater in the higher prostate weight group, but the differences were not significant.

Five patients experienced intraoperative complications, including 3 patients with posterior bladder injury, 1 patient with rectal injury, and 1 patient with ileum injury. In all these patients, the injury was repaired during a robotic operation. No association was observed between prostate weight and incidence of intraoperative complications.

Overall, the rates of postoperative complication were similar across the four groups, as classified according to the Clavien system (Table 2). Bleeding that required transfusion or re-exploration, urinary leakage, and urinary retention were the only complications that were considered to be potentially related to prostate weight. Accordingly, we compared the rates of these complications among the four groups. No significant differences were found.

**Table 2:** Complications and postoperative urinary function outcomes according to prostate weight categories

	**<30**	**≥30, <50**	**≥50, <80**	**≥80**	**P Value**
**Patients (n)**	**17**	**130**	**46**	**6**	
Intraoperative complications	1 (5.9%)	3 (2.3%)	1 (2.2%)	0	0.68
Posterior bladder injury	0	2 (1.5%)	1 (2.2%)	0	
Rectal injury	1 (5.9%)	0	0	0	
Ileum injury	0	1 (0.8%)	0	0	
Overall postoperative complications (Clavien classification)					
I	1 (5.9%)	7 (5.4%)	4 (8.7%)	1 (16.7%)	0.60
II	1 (5.9%)	2 (1.5%)	1 (2.2%)	0	0.68
III	0	2 (1.5%)	1 (2.2%)	0	0.92
Selected postoperative complications					
Urinary retention	0	2 (1.5%)	1 (2.2%)	0	0.92
Urinary leakage	1 (5.9%)	7 (5.4%)	4 (8.7%)	1 (16.7%)	0.45
Bleeding	1 (5.9%)	2 (1.5%)	1 (2.2%)	0	0.92
Urinary continence rate (pad-free rate)					
1 month	52.9%	50.0%	39.1%	33.3%	0.44
3 months	52.9%	51.5%	41.3%	33.3%	0.47
6 months	64.7%	73.8%	65.2%	66.7%	1.04
PSA recurrence					
6 months	1 (5.9%)	8 (6.2%)	3 (6.5%)	0	0.34

### Pathological outcomes

All cases among patients with prostates weighing ≥80 g had pathologic stage T2 (Table 1). However, we found no significant differences in rates of positive margins. Although we noted a trend among the pT3 population, it was not statistically significant. Considering all 219 patients, the mean short-term follow-up was 11.5 months. There were no significant differences in follow-up time between the prostate weight groups.

### Functional outcomes

In the <30 g, 30–49 g, 50–79 g, and ≥80 g groups, 17 (89.5%), 130 (90.9), 46 (90.2%), and 6 (100%) patients had 1, 3 and 6 months of follow-up data, and were therefore eligible for the questionnaire. At 1, 3 and 6 months, the mean extent of return to baseline urinary function was similar across the groups (Table 2). In the group of patients with prostates weighing ≥50 g, the continence rates tended to be somewhat low at 1 and 3 months; however, the continence rates were similar in all the groups after 6 months. Furthermore, at 12 months, the urinary continence rates were similar in the <30 g, 30–49 g, 50–79 g, and ≥80 g groups, specifically being 87.5%, 87.9%, 87.5%, and 66.7%, respectively. The 12-month continence data included 101 patients overall, comprising 8, 66, 24, and 3 patients in the <30 g, 30–49 g, 50–79 g, and ≥80 g groups, respectively. Incontinence events in these groups were thought to be stress urinary incontinence because patients with urge incontinence had been excluded.

### Oncological outcomes

PSA recurrence at 6 months did not differ significantly across the groups.

## Discussion

As RALP has gained acceptance, there has been a continued improvement in the understanding of technical and patient factors that influence perioperative outcomes. Previous case series have investigated the effects of prostate weight on the outcomes of open and laparoscopic radical prostatectomies [3-5]. Large prostates tend to affect operation times, blood loss, transfusion rates, and positive margins. However, although some studies have found significant differences in these quantities, other studies have not.

Huand et al. [16] reported that characteristics of benign prostatic hyperplasia prolong RALP procedures and increase estimated blood loss. However, Huand et al. did not observe any effect of prostate size on positive margins or urinary or sexual function. In the present study, we found no significant differences between the prostate weight groups in terms of transfusion rates, lengths of Foley catheterization, positive surgical margins rates, perioperative complications, or postoperative complications. However, we did find significant differences in total surgical times and robotic times among the prostate weight groups. Larger prostates generally resulted in longer operation times. Additionally, estimated blood loss tended to increase with prostate weight.

However, Zorn et al. [6] reported no significant differences in operative times for patients with greater prostate weights who underwent RALP. Nonetheless, it has also been reported that the prostate median lobe influences RALP operative time [13,17,18]. Sarle et al. [7] specifically reported that RALP was more difficult for patients who had large median lobes. This is a consequence of the base apex approach to RALP, because the median lobe is in the direct view of the laparoscopic camera. Therefore, the median lobe can obscure the posterior border of the prostate and bladder neck, and additionally impair proper identification of the ureteral orifices. It has been reported that both posterior bladder neck and seminal vesicle dissection with an anterior approach require a significantly longer operative time in men with large median lobes [13,19,20]. However, we used a posterior approach to the seminal vesicles; consequently, prostate size did not influence the time required for seminal vesicle dissection. Moreover, our series includes no posterior bladder or rectal injuries during dissection of the bladder neck and seminal vesicle among patients with large median lobes. Therefore, we considered that the posterior approach to the seminal vesicle is effective and safe for patients with large prostates.

In many cases, additional operative time is also required for bladder neck reconstruction in patients with a large median lobe [21]. Indeed, a large prostate and the presence of a median lobe can require additional dissections around the bladder neck, which result in a larger defect that must be anastomosed to the urethra. Depending on the size of the bladder neck and the individual surgeon’s preference, the bladder neck was repaired using either a “fish mouth” or “parachute” technique with anastomosis to the urethral stump [6]. We did observe a trend towards longer anastomosis time among patients with larger prostates; however, the trend was not statistically significant. Because patients with large median lobes often have large prostate glands, either factor may have been responsible for the significantly longer prostate dissection times.

Blood loss requiring transfusion is considered to be the most common intraoperative complication associated with open radical prostatectomy. It has consistently been observed that patients who receive RALP have significantly lower estimated loss and transfusion rates than do patients who receive open radical prostatectomy [4]. In the present study, we have further demonstrated that these benefits of RALP exist irrespective of prostate weight. However, we also observed that blood loss in the large prostate group tended to increase with extended operation times. Nerve-sparing surgery is generally believed to result in greater blood loss. The estimated blood losses experienced by patients who underwent non-nerve-sparing surgeries were 221, 268, 233, and 586 mL in the <30 g, 30–49 g, 50–79 g, and ≥80 g groups, respectively, with the large prostate group (>80 g) experiencing significantly more blood loss than the other groups (P = 0.02). This result suggests that larger prostates are associated with more blood loss.

When investigating the association between prostate weight and surgical outcomes after radical prostatectomy, an important endpoint is the incidence of positive surgical margins rates [4]. In our series, we found that small prostate weight was inversely associated with the risk of positive surgical margins, but the relationship was not statistically significant.

It has been reported that continence rates dependent on various factors, including age, the surgical techniques used, and the surgeon’s experience. Additionally, it has been postulated that neurovascular bundles are displaced posteriorly in larger prostates, and may be obscured by the prostate itself, making them prone to injury. Our results do not support this claim. In our study, prostate size did not have a significant effect on the return of baseline urinary function at either 3 or 6 months (as assessed using validated questionnaires). Although patients with large prostates tended to have lower continence rates at 3 months after surgery, continence rates were similar at 6 months after surgery, irrespective of prostate size. The present study provides the only investigation of this relationship for RALP, although previous studies have confirmed the same finding for open radical prostatectomy and laparoscopic radical prostatectomy.

This study has several limitations. First, there were relatively few patients who had extreme prostate weights (<30 g or ≥80 g), as compared with more common prostate weights. This limited the power of our data analysis for extreme prostate weights. Indeed, among the initial cases, the variation in each case was large. Second, the relatively short mean follow-up of 11.5 months prevented any long-term assessment of patients for biochemical failures and functional outcomes. However, despite the aforementioned limitations, our study provides initial findings regarding the effect of prostate weight on RALP outcomes in Japan.

## Conclusions

In the present study, we demonstrated that operative times for RALP are greater in men with large prostates. We also found that estimated blood losses and anastomosis times tended to be greater in men with large prostates. However, we observed no associations between prostate size and oncological outcomes, urinary continence, or complications, suggesting that RALP can be performed effectively for men with large prostates, despite greater surgical times.

## Abbreviations

RALP: Robot-assisted laparoscopic prostatectomy; PSA: Prostate-specific antigen; CT: Computed tomography; MRI: Magnetic resonance imaging; BMI: Body mass index.

## Competing interests

All authors declare that they have no competing interest.

## Authors’ contributions

TY and KK conceived the study. Data was acquired independently by SS and YH. AO undertook data analysis and interpretation. TY, KT, SK, YU, NK, and YK performed operation. TY and KK prepared the manuscript with contributions from all co-authors. All authors read and approved the final manuscript.

## Authors’ information

TY: lecturer; KT: associate professor; SK: postgraduate doctor; AO: lecturer; KM: lecturer; YU: lecturer; NK: lecturer; SS: associate professor; YH: associate professor; KK: professor and chairman, Department of Nephro-urology, Nagoya City University Graduate School of Medical Sciences. YK: professor and chairman, Department of Urology, Fukushima Medical University School of Medicine.

## Pre-publication history

The pre-publication history for this paper can be accessed here:

http://www.biomedcentral.com/1471-2490/14/6/prepub
